# Low Concentrations of Oxidized Phospholipids Increase Stress Tolerance of Endothelial Cells

**DOI:** 10.3390/antiox11091741

**Published:** 2022-09-01

**Authors:** Christina Mauerhofer, Taras Afonyushkin, Olga V. Oskolkova, Klara Hellauer, Bernd Gesslbauer, Jasmin Schmerda, Yunbo Ke, Andreas Zimmer, Anna A. Birukova, Konstantin G. Birukov, Valery Bochkov

**Affiliations:** 1 Institute of Pharmaceutical Sciences, Department of Pharmaceutical Chemistry, University of Graz, Humboldtstrasse 46/III, 8010 Graz, Austria; 2 Department of Vascular Biology and Thrombosis Research, Center for Physiology and Pharmacology, Medical University of Vienna, Schwarzspanierstrasse 17, 1090 Vienna, Austria; 3 Department of Anesthesiology, University of Maryland School of Medicine, 20 Penn. Street, HSF-2, Room 145, Baltimore, MD 21201, USA; 4 Institute of Pharmaceutical Sciences, Department of Pharmaceutical Technology and Biopharmacy, University of Graz, Universitätsplatz 1/EG, 8010 Graz, Austria; 5 Field of Excellence BioHealth, University of Graz, 8010 Graz, Austria

**Keywords:** oxidized phospholipids, endothelial cell stress, cell survival

## Abstract

Oxidized phospholipids (OxPLs) are generated by enzymatic or autooxidation of esterified polyunsaturated fatty acids (PUFAs) residues. OxPLs are present in circulation and atherosclerotic plaques where they are thought to induce predominantly proinflammatory and toxic changes in endothelial (ECs) and other cell types. Unexpectedly, we found that low concentrations of OxPLs were not toxic but protected ECs from stress induced by serum deprivation or cytostatic drugs. The protective effect was observed in ECs obtained from different vessels and was monitored using a variety of readouts based on different biological and chemical principles. Analysis of the structure–activity relationship identified oxidized or missing fatty acid residue (OxPLs or Lyso-PLs, respectively) as a prerequisite for the protective action of a PL. Protective OxPLs or Lyso-PLs acquired detergent-like properties and formed in solution aggregates <10 nm in diameter (likely micelles), which were in striking contrast with large aggregates (>1000 nm, likely multilayer liposomes) produced by nonoxidized precursor PLs. Because surfactants, OxPLs, and Lyso-PLs are known to extract membrane cholesterol, we tested if this effect might trigger the protection of endothelial cells. The protective action of OxPLs and Lyso-PLs was inhibited by cotreatment with cholesterol and mimicked by cholesterol-binding beta-cyclodextrin but not inactive α-cyclodextrin. Wide-scale mRNA expression analysis in four types of ECs showed the induction of genes encoding for heat shock proteins (HSPs) and secreted prosurvival peptides and proteins. Inducers of HSPs, chemical chaperones, and pure prosurvival factors mimicked the protective action of OxPLs/Lyso-PLs. We hypothesize that oxidation changes the physicochemical properties of PLs, thus promoting membrane cholesterol redistribution or extraction leading to the expression of intra- and extracellular prosurvival factors.

## 1. Introduction

Oxidized phospholipids (OxPLs) are generated by enzymatic or nonenzymatic oxidation of esterified polyunsaturated fatty acids (PUFAs) [1]. These lipids have been studied as both markers and drivers of oxidative stress. Multiple in vitro and in vivo studies have demonstrated important physiological and pathological activities of OxPLs related to innate and acquired immunity, blood clotting, atheroma formation, etc. [2,3,4]. It is currently thought that OxPLs play a mainly toxic and proinflammatory role in disease, although they can also activate selected anti-inflammatory mechanisms [5,6,7]. In recent years, the interest in OxPLs has been boosted by the development of a transgenic animal model expressing antibodies against OxPLs that has generated strong evidence for the causative pathogenic role of OxPLs in atherosclerosis [8], nonalcoholic steatohepatitis [9], ischemia–reperfusion injury [10], and osteoporosis [11]. Furthermore, clinical association studies that were performed in large cohorts of patients have characterized OxPLs as informative biomarkers associated with phenotypes and acute events in cardiovascular disease [12]. These findings suggest that OxPLs and downstream mechanisms potentially may become drug targets, thus justifying the need for a deeper investigation of the biological action of OxPLs at all levels, starting from molecular, membrane, and cellular effects, to the physiological and pathological action in tissues and organs.

The aim of the present work was to study, in more detail, the endothelial toxicity of OxPLs, which are often regarded as toxic substances inducing cell apoptosis [13]. Unexpectedly, we found an activity of OxPLs that had never been characterized before, namely their ability to protect endothelial cells (ECs) from cellular stress induced by biological and chemical factors. Thus, similarly to many other biologically active lipids, OxPLs can induce both protective and toxic effects, which has to be taken into account in the evaluation of the broad biological role of OxPLs.

## 2. Methods

### 2.1. Materials

Phospholipids purchased from Avanti Polar Lipids (Alabaster, AL, USA) were: 1,2-dimyristoyl-*sn*-glycero-3-phosphocholine (DMPC), 1-palmitoyl-2-hydroxy-*sn*-glycero-3-phosphocholine (Lyso-PPC), 1-palmitoyl-2-hydroxy-*sn*-glycero-3-phosphoethanolamine (Lyso-PPE), 1-palmitoyl-2-hydroxy-*sn*-glycero-3-phosphoglycerol (Lyso-PPG), 1-oleoyl-2-hydroxy-*sn*-glycero-3-phosphate (Lyso-OPA), 1-palmitoyl-2-hydroxy-*sn*-glycero-3-phosphoinositol (Lyso-PPI), 1-palmitoyl-2-hydroxy-*sn*-glycero-3-phosphoserine (Lyso-PPS), 1-palmitoyl-2-docosahexaenoyl-*sn*-glycero-3-phosphocholine (PDHPC), 1-palmitoyl-2-arachidonoyl-*sn*-glycero-3-phosphocholine (PAPC), 1-palmitoyl-2-arachidonoyl-*sn*-glycero-3-phosphoserine (PAPS), 1-palmitoyl-2-arachidonoyl-*sn*-glycero-3-phosphatidic acid (PAPA), 1-palmitoyl-2-arachidonoyl-*sn*-glycero-3-phosphoethanolamine (PAPE), 1-palmitoyl-2-arachidonoyl-*sn*-glycero-3-phosphoglycerol (PAPG), and 1-palmitoyl-2-linoleoyl-*sn*-glycero-3-phosphocholine (PLPC). The following lipids were from Cayman Chemicals (Ann Arbor, MI, USA): 1-palmitoyl-2-azelaoyl-*sn*-glycero-3-phosphocholine (PAzPC), 1-palmitoyl-2-(9′-oxononanoyl)-*sn*-glycero-3-phosphocholine (PONPC), 1-palmitoyl-2-(5’-oxovaleroyl)-*sn*-glycero-3-phosphocholine (POVPC), 1-palmitoyl-2-glutaroyl-*sn*-glycero-3-phosphocholine (PGPC), 1-palmitoyl-2-(4-keto-dodec-3-ene-dioyl)-*sn*-glycero-3-phosphocholine (KDdiAPC), 1-palmitoyl-2-(5-keto-6-octene-dioyl)-*sn*-glycero-3-phosphocholine (KOdiAPC), 8-iso-prostaglandin A_2_ (8-iso-PGA_2_), and prostaglandin A_2_. The oxidized lipids OxPAPC, OxPAPS, OxPAPE, OxPAPA, OxPAPG, OxPLPC, and oxidized arachidonic acid (OxAA) were prepared as described [14,15] and stored in chloroform at −80 °C. Just before the experiments, the solvent was removed under a stream of argon with simultaneous vortexing, and the remaining lipid was solubilized in media. The modification of OxPAPC with 20-fold excess of N-acetyl cysteine (NAC, Sigma-Aldrich, St. Louis, MO, USA) in a serum-free cell culture medium M199 was performed as described [16]. Nonelectrophilic oxidized phospholipids PGF_2α_-NH-PC, PGE_2_-NH-PC, 8(OH)C_8_-NH-PC, and 12(OH)C_12_-NH-PC were synthesized as described [17]. Staurosporine was purchased from AppliChem (Darmstadt, Germany). Choline chloride was from Santa Cruz (Dallas, TX, USA); tauroursodeoxycholic acid (TUDCA) and sodium phenyl butyrate were from Sigma-Aldrich (St. Louis, MO, USA). Wortmannin, rapamycin, metformin hydrochloride, doxorubicin hydrochloride, (S)-(+)-camptothecin, U−0126, S3I−201, and geranylgeranylacetone (GGA) were from Selleck Chemicals GmbH (Planegg, Germany). Insulin, MEM amino acids (50×) solution, palmitic acid, and HA15 were from Calbiochem, (San Diego, CA, USA). 2-Aminobicyclo[2.2.1]heptane-2-carboxylic acid (2-amino-2-norbornanecarboxylic acid, BCH), arachidonic acid, cholesterol, methyl-β-cyclodextrin (MβCD), and cytosine β-D-arabinofuranoside hydrochloride (ara-C) were from Sigma Aldrich (St. Louis, MO, USA). Beta-cyclodextrin (β-CD) and alpha-cyclodextrin (α-CD) were purchased from Thermo Fisher Scientific (Waltham, MA, USA). Recombinant human HB-EGF (Sigma-Aldrich, St. Louis, MO, USA), VEGF (ImmunoTools, Friesoythe, Germany), IL-11 (eBioscience, San Diego, CA, USA), VIP (Sigma-Aldrich, St. Louis, MO, USA), and SCF (ImmunoTools, Friesoythe, Germany) were used according to manufacturers’ instructions. 

### 2.2. Analyses of Cells Accumulated in BAL

Male, wild-type, 8-week-old C57BL/6J mice were divided into 3 groups: control group (6 mice/group), doxorubicin group (6 mice/group), and doxorubicin + OxPAPC group (7 mice/group). The mice were injected i.p. with doxorubicin (8 mg/kg) 3 times on alternate days. OxPAPC was injected i.m. at a dose of 1 mg/kg, 3 times on alternate days. Administration of OxPAPC immediately followed the injection of doxorubicin. About 24 h after the last injection of doxorubicin or doxorubicin + OxPAPC, the mice were arrested by anesthesia (ketamine 100 mg/kg and xylazine 10 mg/kg). BALs were collected and the BAL cells were stained with a calcein/propidium iodide cell stain double stain kit (Sigma, St. Louis, MO, USA). The inflammatory cells were analyzed under a fluorescent microscope EVOS FL Auto 2 (AMAFD2000 from EVOS Cell Imaging Systems via Thermo Fisher Scientific, Waltham, MA, USA). The number of dead cells was expressed as % of total cells detected. All the protocols involving animal care and treatment procedures were approved by the Institutional Animal Care & Use Committee of the University of Maryland.

### 2.3. Culturing of Cells

HUVECtert cells were provided by the Institute of Immunology, Medical University of Vienna, Austria. HUVEC (Lonza, Basel, Switzerland) and HUVECtert cells were cultured in Gibco M199 medium with Earl’s salts (Thermo Fisher Scientific, Waltham, MA, USA) supplemented with 20% fetal calf serum (FCS, Sigma-Aldrich, St. Louis, MO, USA), 2% glutamine (Lonza, Basel, Switzerland), penicillin–streptomycin–fungizone (Lonza, Basel, Switzerland) and ECGS/H (PromoCell, Heidelberg, Germany). Primary HUVECs were cultured up to passage 5. HPAECs (Lonza) were grown in EBM-2 medium (Lonza, Basel, Switzerland) supplemented with EGM-2 SingleQuot kit (Lonza, Basel, Switzerland), 10% fetal bovine serum (FBS, Thermo Fisher Scientific, Waltham, MA, USA), and penicillin–streptomycin–neomycin (Thermo Fisher Scientific, Waltham, MA, USA). HLMVEC cells (Lonza, Basel, Switzerland) were cultured up to passage 9 in the endothelial cell basal medium MV (PromoCell, Heidelberg, Germany) supplemented with endothelial cell growth medium MV SupplementPack (Promocell, Heidelberg, Germany), penicillin–streptomycin–neomycin and 10% FBS (both from Thermo Fisher Scientific, Waltham, MA, USA). HBMVEC cells (Lonza, Basel, Switzerland) were grown up to passage 9 in the medium kit without serum but in the presence of Culture Boost (CellSystems, Troisdorf, Germany) and penicillin–streptomycin–neomycin (Thermo Fisher Scientific, Waltham, MA, USA). Endothelial cells were cultured in 75 cm^2^ Nunc cell culture flasks (Thermo Fischer Scientific, Waltham, MA, USA) in a humidified atmosphere with 5% CO_2_ at 37 °C. After reaching 80% confluency, the cells were seeded into Nunclon Delta surface plates (Thermo Fisher Scientific, Waltham, MA, USA) for cell survival/toxicity/Western blot analyses experiments or gold electrodes for transendothelial electrical resistance measurement.

### 2.4. Nuclei Counting Using Hoechst 33,342

After the treatment of cells, supernatants were removed and 1 µg/mL Hoechst 33,342 (Thermo Fisher Scientific, Waltham, MA, USA) in media with 0% serum was added. After 20 min of incubation at 37 °C, the fluorescently labeled nuclei of attached cells were counted using a multi-mode plate reader EnSight (PerkinElmer, Waltham, MA, USA). 

### 2.5. Metabolic Activity Assessment

An XTT assay was performed using tetrazolium salt 2,3-bis-(2-methoxy-4-nitro-5-sulfophenyl)-2*H*-tetrazolium-5-carboxanilide (XTT, Thermo Fisher Scientific, Waltham, MA, USA) and phenazine methosulfate (PMS, Thermo Fisher Scientific, Waltham, MA, USA). After the incubation of cells with a mixture of 0.2 mg/mL XTT and 0.015 mg/mL PMS in medium with 0% FCS for 2 h, absorbance at 450 nm was measured.

ATP assay and LDH assays were performed using an ATPlite assay kit (PerkinElmer, Waltham, MA, USA) or Cytotoxicity Detection Plus kit (Roche, Basel, Switzerland), respectively, according to the manufacturers’ instructions. Caspase-3 activation was measured by a Caspase Glo assay kit from Promega (Fitchburg, WI, USA).

### 2.6. ELISA

The determination of proteins in the cell culture medium was performed using the following ELISA kits according to the manufacturers’ instructions: VIP (USCN Life Science Inc., Wuhan, China), IL-11 (RayBiotech, Peachtree Corners, GA, USA), HB-EGF (RayBiotech, Peachtree Corners, GA, USA), Platinum VEGF ELISA kit (eBioscience, San Diego, CA, USA), and SCF (R&D Systems, Minneapolis, MN, USA).

### 2.7. Proteomic Analyses of Secreted Proteins

#### 2.7.1. Sample Preparation

HUVECtert cells were treated with OxPAPC (25 µg/mL) in serum-free M199 medium in 12-well plates. After 24 h, the conditioned medium (CM) of three single wells from control cells and OxPAPC-treated cells was pooled, respectively. After centrifugation at 17,000× *g* for 20 min, supernatants were diluted with PBS to a final volume of 12 mL and centrifuged at 100,000× *g* for 60 min. Supernatants (10 mL) were concentrated and rebuffered to 50 mM NH_4_HCO_3_ using Amicon centrifugal filters (Merck KGaA, Darmstadt, Germany) with an MWCO of 3 kDa (final volume: 120 µL). Protein concentration was determined with the MicroBCA Protein Assay Kit (Thermo Fisher Scientific, Waltham, MA, USA).

#### 2.7.2. In-Solution Protein Reduction, Alkylation, and Digestion

Samples were incubated with 0.04 µg dithiothreitol (DTT, Sigma-Aldrich, St. Louis, MO, USA) per µg protein at 56 °C for 30 min. After incubation with 0.2 µg iodoacetamide (IAA, Sigma-Aldrich, St. Louis, MO, USA) per µg protein for 30 min at RT in the dark, the reaction was quenched with 0.2 µg DTT per µg protein. Sequencing-grade trypsin (1 µg per 75 µg protein, Promega, Fitchburg, WI, USA) was added and samples were incubated for 4 h at 37 °C. After a further aliquot of trypsin (1 µg per 75 µg protein) was added, the samples were incubated at 37 °C overnight. The digestion was stopped by acidifying the samples to a pH of 2.5 by adding trifluoroacetic acid (Thermo Fisher Scientific, Waltham, MA, USA).

#### 2.7.3. Analysis by NanoLC-MSMS 

NanoLC-MSMS analysis was performed on an UltiMate 3000 RSLCnano system (Thermo Fisher Scientific, Waltham, MA, USA) online coupled to a LTQ XL mass spectrometer (Thermo Fisher Scientific, Waltham, MA, USA) as described previously [18] with the following adaptations. For separation, an Acclaim PepMap RSLC column (C18, 75 µm × 50 cm, 2 µm, 100 Å; Thermo Fisher Scientific, Waltham, MA, USA) was used. The flow rate of the nano-HPLC system was set at 200 nL/min. The mobile phases were: (A) 99.9% (*v*/*v*) water (HPLC grade, VWR International, Radnor, PA, USA) and 0.1% (*v*/*v*) formic acid (VWR International, Radnor, PA, USA); and (B) 80% (*v*/*v*) acetonitrile (HPLC grade, VWR International, Radnor, PA, USA) and 0.08% (*v*/*v*) formic acid. The HPLC gradient for separation was 4% B for 10 min, 4–40% B in 480 min, 40–90% B in 5 min, 90% B for 10 min, and 4% B for 20 min. 

For peptide identification, RAW files were converted into MGF files using ProteoWizard [19] and analyzed using the MASCOT search engine (Matrix Science, London, UK). All MS/MS spectra were searched against the SwissProt protein sequence database as described previously [18]. The results were filtered to peptide scores ≥30 and to a 1% false discovery rate using Mascot. The exponentially modified protein abundance index (emPAI) score [20], which is automatically calculated by MASCOT, was used to determine differences in protein abundance.

### 2.8. Transendothelial Electrical Resistance

To HUVEC cells grown on gold electrodes in M199 medium with 20% FCS, 2% glutamine, penicillin–streptomycin–fungizone, and ECGS/H, OxPAPC (5 µg/mL) was added in 0% serum. The measurement of transendothelial electrical resistance was performed using an electrical cell–substrate impedance sensing system (ECIS, Applied Biophysics, Troy, NY, USA) as described [21,22].

### 2.9. Dynamic Light Scattering

The particle size of phospholipids was determined by dynamic light scattering with a Zetasizer Nano ZS from Malvern Instruments (Malvern Instruments, Malvern, UK). Measurements were performed in UV cuvettes (Brand, Wertheim, Germany) at 37 °C with back-scattering detection at 173°. Lipids were prepared with a concentration of 100 µM in serum-free and phenol-red-free M199 medium (Gibco via Thermo Fisher Scientific, Waltham, MA, USA). Each measurement was performed in triplicate.

### 2.10. Western Blotting

After the indicated treatments, cells were washed once with precooled DPBS (Thermo Fisher Scientific, Waltham, MA, USA). Subsequently, radioimmunoprecipitation assay (RIPA) buffer (Sigma-Aldrich, St. Louis, MO, USA) supplemented with Roche cOmplete protease inhibitor cocktail (Sigma-Aldrich, St. Louis, MO, USA) and PhosStop (Roche, Basel, Switzerland) was added. After incubation on ice for 10 min, the cells were scraped off and transferred into 1.5 mL vials. Samples were diluted to obtain equal protein concentrations according to MicroBCA assay results by mixing with different volumes of RIPA buffer. Prior to SDS-PAGE, samples were mixed with 4x Laemmli sample buffer (BioRad, Hercules, CA, USA) supplemented with 10% β–mercaptoethanol (Roth, Karlsruhe, Germany) and heated for 10 min at 95 °C.

After determination of protein concentrations using a MicroBCA Protein Assay Kit, protein samples were separated by 10% SDS-PAGE and blotted onto a nitrocellulose membrane (Pall, NY, USA). The transfer was carried out in a Mini-PROTEAN^®^ Tetra Vertical Electrophoresis Cell (BioRad, Hercules, CA, USA) using Towbin buffer (192 mM glycine, 25 mM TRIS (pH 8.4)) and 20% methanol at the constant voltage (100 V) for 1 h at 4 °C. Efficiency of the transfer was checked by Ponceau S staining (Sigma-Aldrich, St. Louis, MO, USA). Unspecific antibody binding was prevented by blocking the membrane with 3% BSA in TBST (BioRad, Hercules, CA, USA) for 30 min at room temperature. Membranes were incubated with primary antibodies at different dilutions (Table 1) in TBST/1% BSA overnight at 4 °C. Then, the membranes were washed three times for 5 min with TBST buffer and incubated in TBST for 1 h at room temperature with HRP-conjugated secondary antibodies (goat anti-rabbit HRP-AB diluted 1:3000 from Cell Signaling Technology Europe (Leiden, The Netherlands) or anti-mouse-HPR-AB from SantaCruz (Santa Cruz, CA, USA) (diluted 1:8000). After 3 washing steps with TBST for 5 min each, membranes were incubated with HRP substrate Amersham ECL Select (GE Healthcare, Chicago, IL, USA) for 3 min and chemiluminescence was detected by G::Box (Syngene, Cambridge, UK). 

### 2.11. Statistical Analyses

All data are presented as means ± SD. Single comparisons were evaluated by Student’s *t*-test. Multiple comparisons with one control group were assessed by one-way ANOVA with post-hoc Dunnett’s multiple comparisons test. *p* values of less than 0.05 were considered as statistically significant. * *p* ≤ 0.05, ** *p* ≤ 0.01, and *** *p* ≤ 0.001.

## 3. Results

### 3.1. Phenomenon of Endothelial Protection by OxPLs

#### 3.1.1. Protective Action of OxPLs Can Be Demonstrated Using Different Experimental Readouts

**Figure 1:**
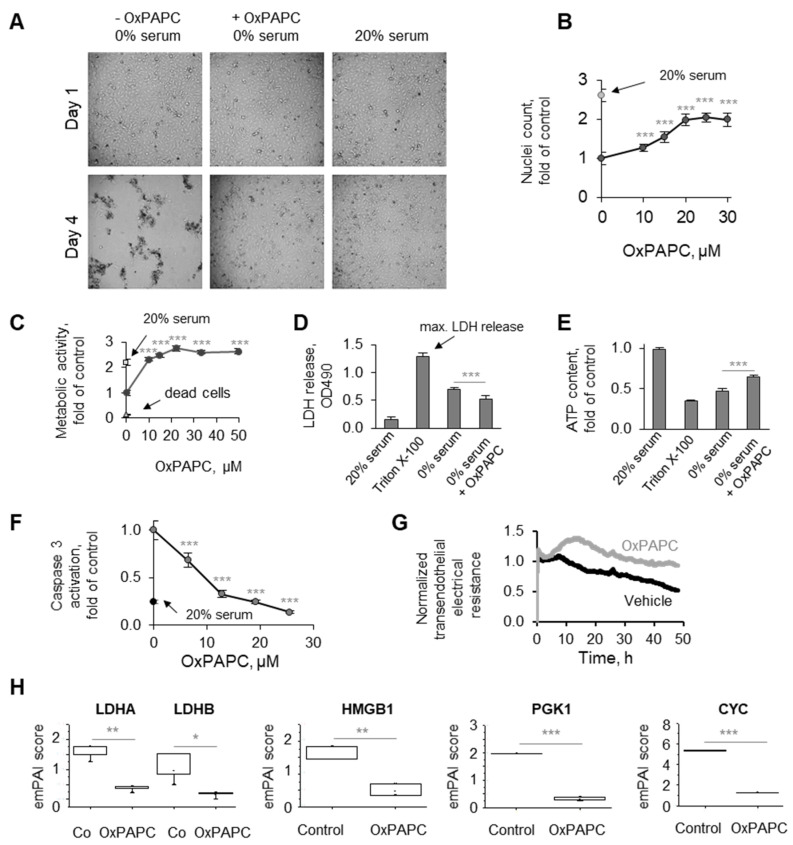
**OxPAPC protects endothelial cells from serum deprivation.** (**A**–**H**) HUVECtert cells were incubated in serum-free medium 199 in the presence or absence of indicated concentrations of OxPAPC. After 48 or 72 h, cell survival was estimated by phase-contrast microscopy (**A**), counting of nuclei of attached cells (**B**), metabolic activity (XTT assay, **C**), release of LDH (**D**), quantification of cellular ATP (**E**), caspase activity (**F**), or endothelial monolayer electrical resistance (**G**). Panel (**H**) presents proteomics data obtained by nanoLC/MS analysis showing relative abundancy (emPAI score) of four intracellular proteins in the incubation medium. Technical details are described in the Section 2. * *p* ≤ 0.05, ** *p* ≤ 0.01, and *** *p* ≤ 0.001.

During our studies of biological activities of OxPLs in ECs, we made an unexpected observation that OxPLs protected HUVECtert from cell death induced by serum deprivation. The better condition of OxPAPC-treated cells as compared to cells without OxPAPC can be easily recognized under the phase contrast microscope without any specific staining (Figure 1A). The protective action of OxPAPC was confirmed using several readouts based on different biological, chemical, and physical principles. These included the counting of nuclei of attached cells using image analysis (Figure 1B), analysis of the metabolic activity of attached cells (Figure 1C), quantification of LDH released into the medium using an enzymatic method (Figure 1D), analysis of cellular ATP levels (Figure 1E), an assay for the activity of caspase 3 (Figure 1F), analysis of the cell–cell barrier based on electrical properties of the monolayer (Figure 1G), or analysis by mass spectrometry of the typical intracellular proteins released into the culture medium (Figure 1H). All assays consistently demonstrated the ability of OxPAPC to protect cells from damage induced by serum deprivation.

#### 3.1.2. OxPLs Protect Primary Endothelial Cells Isolated from Different Vessels

**Figure 2:**
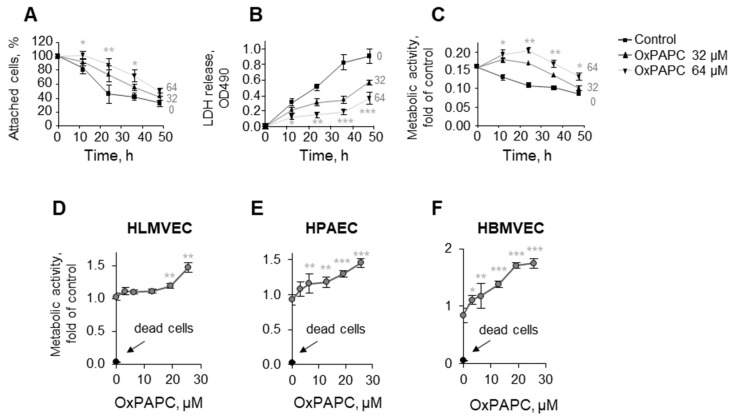
**OxPAPC protects different primary endothelial cell types from serum-deprivation-induced cell death.** (**A**–**C**) Primary HUVECs were incubated for 48 h in 2% serum and then attached cell counts (**A**), levels of LDH released into the medium (**B**), or metabolic activity (XTT) (**C**) were determined. Measurement of the metabolic activity (XTT assay) in primary microvascular ECs from human lung (**D**), brain (**F**), or macrovascular ECs from human pulmonary artery (**E**) was performed after 48 h of incubation in the absence or presence of indicated concentrations of OxPAPC. * *p* ≤ 0.05, ** *p* ≤ 0.01, and *** *p* ≤ 0.001.

The experiments described above were performed on immortalized HUVECtert and, therefore, we tested if OxPAPC can also protect primary ECs. The lipid prevented the death of primary HUVECs as assessed by the counting of attached cells, LDH release, and metabolic activity (Figure 2A–C). In addition to HUVECs, OxPAPC protected primary microvascular ECs isolated from the human lung and brain, as well as macrovascular endothelial cells from the pulmonary artery from serum deprivation (Figure 2D–F). In summary, our data show that the phenomenon of protection is not limited to immortalized HUVECtert but is observed in primary HUVECs and ECs isolated from other vessels.

#### 3.1.3. Pretreatment with OxPLs Does Not Inhibit Proliferation of ECs after Serum Replenishment

**Figure 3:**
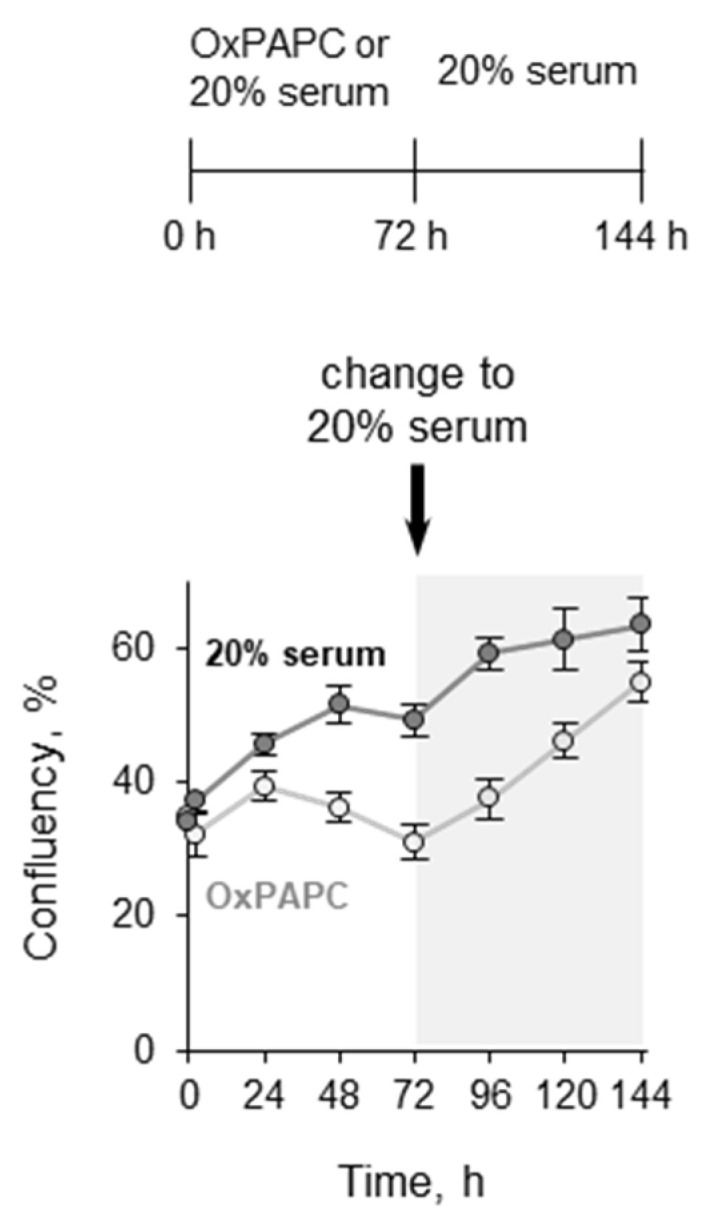
**OxPAPC treatment does not reduce the proliferation capacity of cells.** The confluency of HUVECtert cells incubated with 20 µM OxPAPC under serum-free conditions or without OxPAPC under 20% serum was measured every 24 h. After 72 h, the medium in all wells was exchanged to that containing 20% serum, and measurements were continued. As a control, cells growing in medium with 20% serum were analyzed at the same time points.

OxPAPC contains electrophilic molecules which potentially can damage DNA, stop proliferation, and induce cellular senescence. To test if OxPAPC protects cells in the short term but induces long-term loss of proliferative potential, we pretreated HUVECtert cells with OxPAPC in serum-free medium for 72 h followed by a switch to medium containing 20% serum (Figure 3, lower curve). Cells that were incubated with 20% serum throughout the experiment served as a control (upper curve). The cell confluency in the OxPAPC-pretreated wells rapidly increased in the next days after the readdition of serum-containing medium. The result shows that, at least within the tested limited number of cell divisions, OxPAPC-treated cells did not lose the ability to proliferate.

#### 3.1.4. OxPAPC Protects Cells in the Presence of Toxins

**Figure 4:**
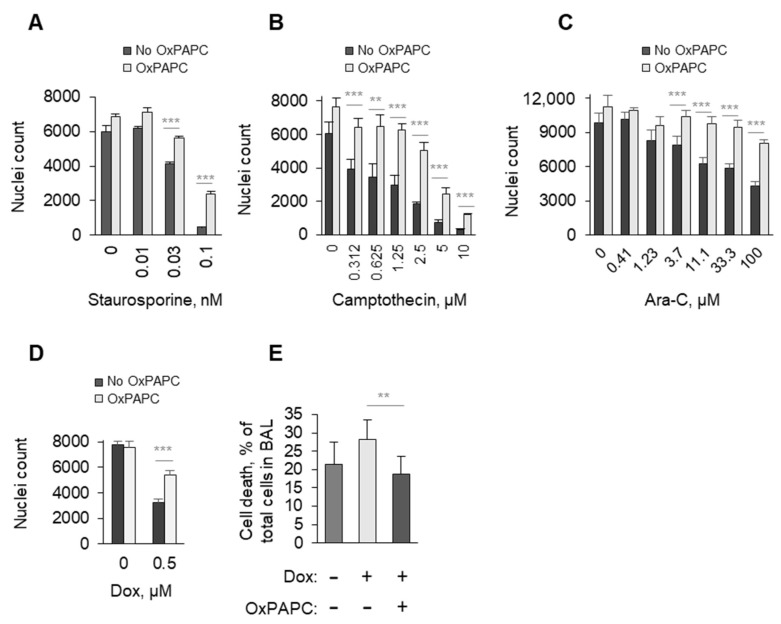
**Protective effect of OxPAPC against cytostatic drugs in serum-containing medium.** HUVECtert cells were incubated with OxPAPC in the presence of serum (7 to 12%) and indicated concentrations of staurosporine (**A**), camptothecin (**B**), arabinoside-C (**C**), or doxorubicin (**D**) for 48 h, followed by counting of Höchst-stained nuclei of remaining attached cells. (**E**) Wild-type mice were injected i.p. with doxorubicin (8 mg/kg) and/or i.m. OxPAPC (1 mg/kg) three times in 5 days. 24 h after last injection, BAL samples were collected. Rates of cell death in lavage were determined upon dual staining with calcein and propidium iodide. Number of dead cells are expressed as % of total counted cells. ** *p* ≤ 0.01, and *** *p* ≤ 0.001.

We further tested whether OxPAPC can improve the survival of ECs (HUVECtert) in the presence of toxins. To this end, the cells were treated with drugs inducing cell death through various biological mechanisms such as staurosporine, camptothecin, arabinoside C, and doxorubicin (Figure 4A–D). The number of cells surviving in the presence of OxPAPC was higher as compared to samples treated with the toxins alone. Furthermore, in an in vivo model of doxorubicin-induced lung toxicity (i.v. application), we observed a reduction in dead cells in bronchoalveolar lavage after cotreatment with OxPAPC (i.m. application) (Figure 4E). In sum, the data show that OxPAPC protected cells from serum deprivation and cytostatics.

### 3.2. Potential Mechanisms Mediating Protective Action of OxPLs 

**Figure 5:**
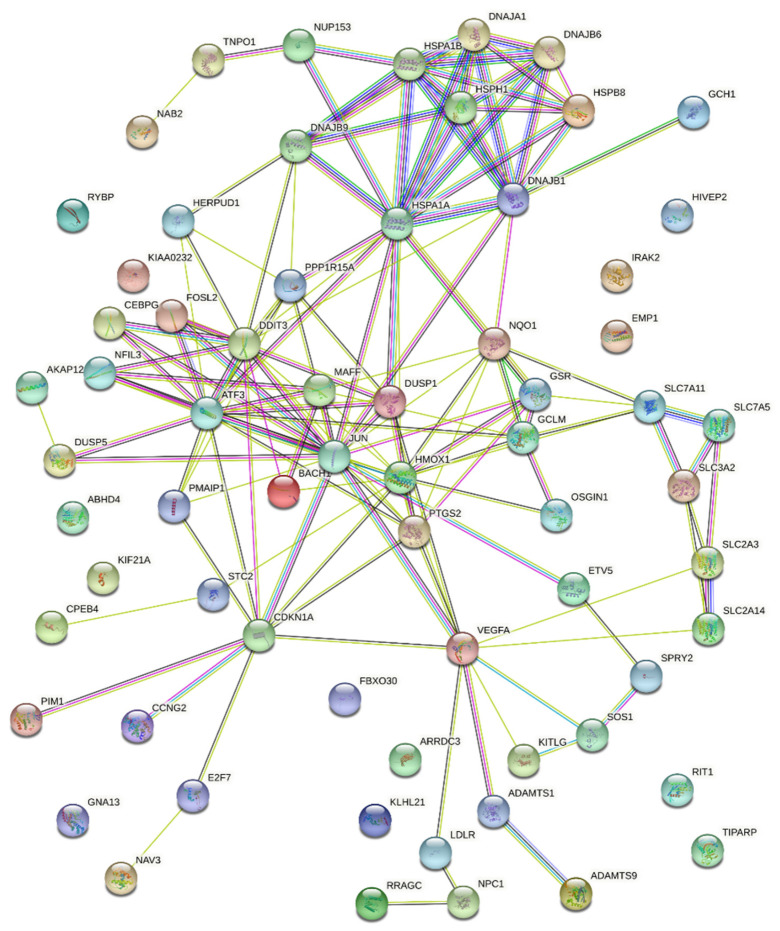
**STRING protein–protein association network.** Microarray and RNAseq analyses were used to identify proteins that were upregulated by OxPAPC in different types of ECs (HAECs, HCAECs, HUVECtert and HPAECs). The network shows only genes that were upregulated in at least three EC types.

We applied wide-scale mRNA expression analysis in order to identify the possible signaling pathways mediating protection. In order to focus on the most relevant gene expression changes, we selected messages that were reproducibly upregulated by OxPAPC in four types of ECs that were analyzed in one published (HAECs [23]) and three of our own experiments: HUVECtert and HCAECs (Affymetrix microarrays) and HPAECs (RNAseq). For further analysis, we selected those 66 mRNAs that were upregulated in either four or three EC types. The STRING network and functional enrichment analysis for 66 messages are presented in Table 2 and Figure 5. The most represented GO terms, pathways and clusters were cell stress (GO:0033554; HSA-2262752), unfolded proteins and UPR (GO:0006986; GO:0006457; HSA-381119), NRF2 (WP2884), heat shock factor and heat shock proteins (HSA-3371453; HSA-3371571), and amino acid transport (CL:14971). Potentially relevant for protection was also VEGF-VEGFR signaling (WP3888). All these hits were statistically significant as documented by false identification rate values of 3 × 10^−8^ to 8 × 10^−3^. Analysis of 26 genes that were downregulated by OxPAPC in at least three endothelial cell lines did not point to any potential mechanism that could explain the protective action of this lipid (data not shown).

The analysis of genes directly involved in pro- or antisurvival signaling did not reveal a clear hint pointing to the mechanisms of protection. Approximately similar numbers of genes positively or negatively associated with cell death were upregulated by OxPAPC (GO:0043068 and GO:0060548, 15 and 19 proteins, respectively). However, several genes (*HMOX1*, *DUSP1*, *NQO1*, *PTGS2*, *JUN*, *DNAJA1* and *CDKN1A*) are annotated to have both pro- and antiapoptotic properties which may be cell-type-dependent. In endothelial cells, some of these genes (e.g., *HMOX1* and *NQO1*) are known to have protective effects [24]. In summary, because of the lack of strong and unequivocal prosurvival hits, we hypothesized that the protective action may be a net effect of several pathways each promoting cell survival. Based on the gene expression pattern, the likely candidate mechanisms were antioxidant defense, protection of amino acid uptake and proteostasis, as well as the secretion of growth factors. These possibilities were tested experimentally as described in the following paragraphs.

**Figure 6:**
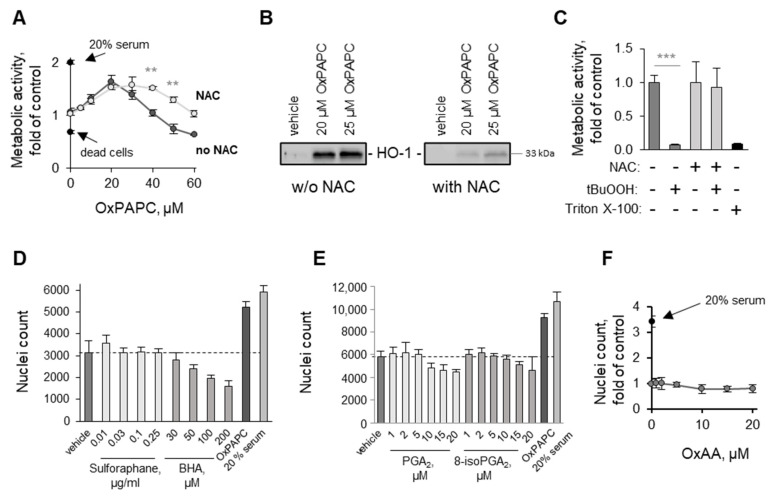
**Electrophilic groups present in OxPAPC are not responsible for protective effects in endothelial cells.** (**A**) OxPAPC was preincubated with a 30-fold molar excess of N-acetyl cysteine (NAC) for 30 min at 37 °C and then added to HUVECtert cells. Cells were incubated for 48 h in serum-free medium, and the metabolic activity was measured using an XTT assay. Protective effect of OxPAPC was not reversed by NAC. In contrast, induction of HO-1 by OxPAPC and toxic effects of tBuOOH (75 μM) were inhibited by 1 mM NAC, which confirms antioxidant properties of NAC (**B**,**C**). (**D**–**F**), Treatment with electrophilic compounds sulforaphane (**D**), butylated hydroxyanisole (BHA) (**D**), prostaglandin A_2_ (PGA_2_) (**E**), 8-iso-prostaglandin A_2_ (8-iso-PGA_2_) (**E**), or oxidized arachidonic acid (OxAA) (**F**) did not protect HUVECtert cells against serum deprivation. Cell survival was measured by counting Höchst-stained nuclei of attached cells after 48 h. Dashed lines correspond to control (vehicle) treatment. ** *p* ≤ 0.01, and *** *p* ≤ 0.001.

Several genes presented in Table 2 are regulated by NRF2 that is well known for its cell-protective properties. We tested if this transcription factor may be the major driver of endothelial protection induced by OxPAPC, which is known to activate NRF2 [16]. The treatment of cells with OxPAPC in the presence of N-acetyl cysteine (NAC), a chemical trap for electrophilic molecules and precursor of glutathione, did not reduce the maximal protection of ECs at the optimal protective OxPAPC concentration (20 µM OxPAPC in Figure 6A). The efficiency of NAC as a nucleophilic chemical trap and antioxidant was confirmed under the same experimental conditions by (i) reduced induction by OxPAPC of NRF2-dependent protein HO-1 (Figure 6B) and (ii) protection by NAC from toxicity induced by a lipid hydroperoxide (Figure 6C). Interestingly, NAC reduced the toxicity of high concentrations of OxPAPC (above 30 µM in Figure 6A). Taken together, the data suggest that electrophilic molecules present in OxPAPC play no or a minimal role in protection but are important causes of toxicity at higher concentrations of OxPAPC. As an independent approach to characterize the involvement of NRF2, we tested the electrophilic compounds sulforaphane, butylated hydroxyanisol (BHA), PGA_2_, and 8-isoPGA_2_. They did not improve cell survival, thus suggesting that activation of NRF2 by electrophiles was not a key mechanism of the protective activity (Figure 6D,E). In addition, oxidized free (nonesterified) arachidonic acid (OxAA), which is expected to contain similar electrophilic peroxidation products to OxPAPC, also did not improve the survival of serum-deprived ECs (Figure 6F). Finally, as will be shown below, also nonelectrophilic OxPLs (e.g., PGPC) and other nonelectrophilic molecules (β-cyclodextrins) were able to protect ECs as efficiently as OxPLs. In summary, the data show that the protective action is neither mimicked by electrophilic molecules known to activate NRF2, nor inhibited by a nucleophilic trap NAC. Thus, electrophilic molecular species do not play a significant role in the endothelium-protective action of OxPAPC.

**Figure 7:**
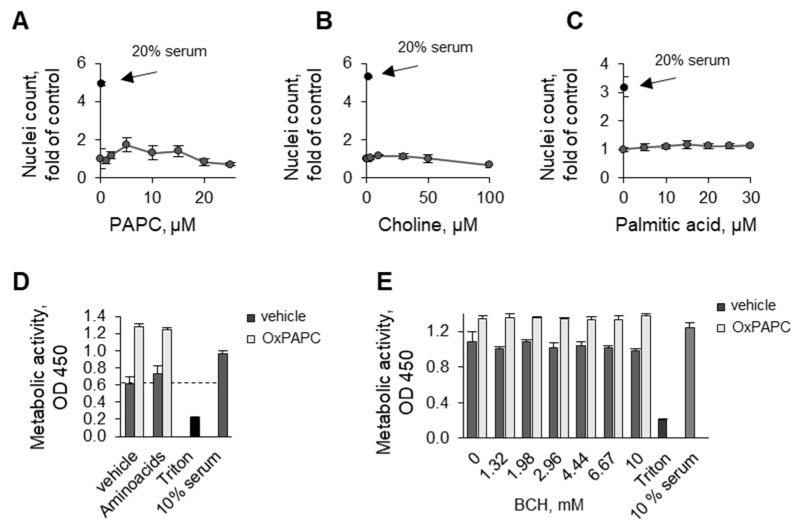
**Supply of lipids and amino acids does not play a mechanistic role in protective action of OxPAPC.** HUVECtert cells were incubated with increasing concentrations of PAPC (**A**), choline (**B**), or palmitic acid (**C**) for 48 h, followed by counting Höchst-stained nuclei of attached cells. (**D**) HUVECtert cells were incubated with serum-free medium in the presence or absence of an excess of essential amino acids (1×) and OxPAPC. After 72 h an XTT assay was performed. Dashed line shows levels from control (vehicle-treated) cells. (**E**) HUVECtert cells were treated with an aminoacid transporter inhibitor 2-amino-2-norbornanecarboxylic acid (BCH) and OxPAPC for 48 h followed by analysis of metabolic activity by the XTT assay.

The simplest mechanism of protective action of OxPAPC could be the delivery of building blocks (choline and fatty acids) for the synthesis of phospholipids that are necessary for membrane repair in serum-deprived cells. However, the supplementation of cells with (i) nonoxidized PLs, (ii) physiological concentrations of choline in addition to that present in cell culture medium, or iii) palmitic acid did not protect cells (Figure 7A–C). Furthermore, as will be described below, compounds with non-phospholipid structures (β-cyclodextrins), which do not contain building blocks for phospholipids, nevertheless potently protected ECs similarly to OxPLs. Thus, the role of OxPLs as precursors for the replenishment of intracellular PLs is an unlikely mechanism of protective action.

Another possible consequence of serum deprivation is an impairment of amino acid uptake. It has been shown that the lack of growth factors downregulated transporters of essential amino acids, thus leading to the deficit of intracellular amino acids in spite of their abundant presence in cell culture medium [25]. Previously published results of microarray analysis in human aortic endothelial cells [26], as well as our unpublished results of microarray, proteomics, and RNAseq experiments, demonstrated upregulation by OxPAPC of multiple membrane transporters. In particular, experiments performed in HPAECs and HUVECtert cells demonstrated the induction of SLC7A5 and SLC3A2 (Table 2), which form an uptake system for essential amino acids such as L-leucine, which activates the major regulator of cell proliferation and survival, mTOR [27]. Based on these findings, we hypothesized that the protective action of OxPAPC may result from the upregulation of membrane transporters leading to the replenishment of essential amino acids, which in turn stimulate prosurvival mTOR activity and normalize protein and glutathione synthesis, i.e., triggering events that finally protect cells from serum deprivation. However, experimental data did not support this hypothesis. We found that the supplementation of cells with an excess of amino acid mixture had no effect on cell survival either in the presence or absence of OxPAPC (Figure 7D). Furthermore, an inhibitor of SLC7A5/SLC3A2 transporter 2-aminobicyclo[2.2.1]heptane-2-carboxylic acid (BCH) did not reverse the protective action of OxPAPC (Figure 7E). Thus, the restoration of the intracellular amino acid pool via the upregulation of membrane amino acid transporters is an unlikely explanation for the protective activity of OxPAPC.

**Table 2:** Effects of OxPAPC on gene expression: Functional enrichment analysis using STRING database including Gene Ontology, Reactome, Local network cluster and WikiPathway.

	GO-Term/Pathway/Cluster	False Discovery Rate	Genes
Stress response	GO:0033554; Cellular response to stress	3.19 × 10^−7^	PPP1R15A; HMOX1; GSR; DNAJB9; DNAJB1; IRAK2; CPEB4; STC2; SLC7A11; HSPB8; VEGFA; ETV5; NQO1; E2F7; PMAIP1; ATF3; PTGS2; JUN; RRAGC; HSPA1B; HSPA1A; BACH1; CDKN1A; HERPUD1; DDIT3
	HSA-2262752; Cellular responses to stress	9.87 × 10^−8^	PPP1R15A; GSR; DNAJB1; DNAJB6; HSPB8; VEGFA; CEBPG; HSPH1; ATF3; JUN; RRAGC; HSPA1B; DNAJA1; CDKN1A; NUP153; DDIT3
NRF2	WP2884; NRF2 pathway	3.53 × 10^−8^	SLC2A3; HMOX1; GSR; DNAJB1; SLC7A11; NQO1; MAFF; GCLM; HSPA1B; SLC2A14
UPR	GO:0006986; Response to unfolded protein	4.61 × 10^−8^	DNAJB9; DNAJB1; STC2; HSPB8; HSPH1; ATF3; HSPA1B; HSPA1A; DNAJA1; HERPUD1; DDIT3
	GO:0006457; Protein folding	0.0081	DNAJB1; DNAJB6; HSPH1; HSPA1B; HSPA1A; DNAJA1
	HSA-381119; Unfolded protein response (UPR)	0.0055	DNAJB9; CEBPG; ATF3; HERPUD1; DDIT3
HSF1	HSA-3371453; Regulation of HSF1-mediated heat shock response	0.0019	DNAJB1; DNAJB6; HSPB8; HSPH1; HSPA1B; NUP153
Cell death *	GO:0060548; Negative regulation of cell death	8.50 × 10^−7^	HMOX1; KITLG; DUSP1; VEGFA; DNAJB6; CPEB4; NPC1; SLC7A11; NQO1; PTGS2; GCLM; JUN; PIM1; HSPA1B; HSPA1A; SPRY2; DNAJA1; CDKN1A; HERPUD1
	GO:0043066; Negative regulation of apoptotic process	5.19 × 10^−6^	HMOX1; KITLG; DUSP1; VEGFA; DNAJB6; CPEB4; NQO1; PTGS2; GCLM; JUN; PIM1; HSPA1B; HSPA1A; SPRY2; DNAJA1; CDKN1A; HERPUD1
	GO:0043068; Positive regulation of programmed cell death	5.19 × 10^−6^	HMOX1; DUSP1; NQO1; PMAIP1; ATF3; OSGIN1; PTGS2; JUN; DNAJA1; AKAP12; CDKN1A; SOS1; GNA13; RYBP; DDIT3
Amino acid transport	CL:14971; Amino acid transport across the plasma membrane	0.00090	SLC2A3; SLC7A5; SLC7A11; SLC3A2; SLC2A14
	GO:0089718; Amino acid import across plasma membrane	0.0039	SLC7A5; SLC7A11; SLC3A2
VEGFA **	WP3888; VEGFA-VEGFR2 signaling pathway	5.81 × 10^−5^	VEGFA; DNAJB9; ADAMTS1; PTGS2; DUSP5; JUN; HSPA1B; DNAJA1; HERPUD1; ADAMTS9; SLC2A14

False Discovery Rate: Shown are *p*-values corrected for multiple testing within each category using the Benjamini–Hochberg procedure. * HMOX1, DUSP1, NQO1, PTGS2, JUN, DNAJA1 and CDKN1A are annotated to have both pro- and antiapoptotic properties, based on GO terms. HMOX1 showed proapoptotic effects only in malignant cells but not in healthy cells (reviewed in [28]). NQO1 is an antioxidant enzyme which has cell-protective effects [29]. ** In wide-scale mRNA expression analysis, VEGFA was found to be upregulated in 2 out of 4 cell types, which is below the “induction in minimum 3 out of 4 mRNA profiling experiments” rule applied for other genes. However, we have multiple additional data showing that OxPAPC upregulates VEGFA in endothelial cells [30]. Thus, VEGFA was included in STRING analysis.

**Figure 8:**
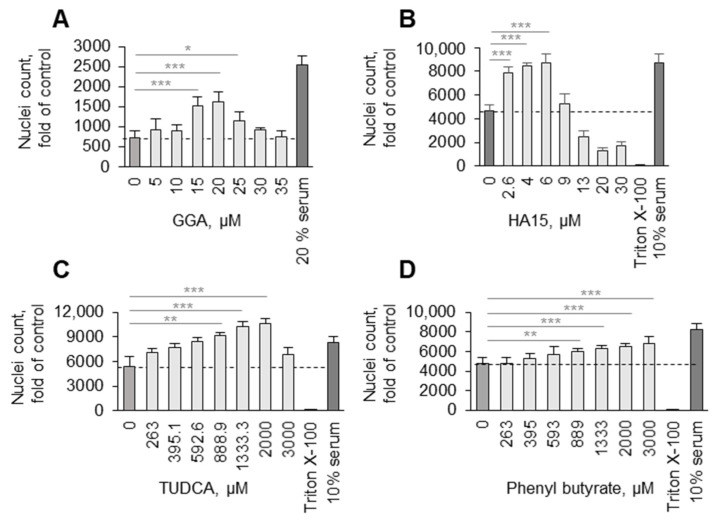
**Inducers of heat shock protein expression and chemical chaperones protect HUVECs from serum deprivation.** (**A**,**B**) HUVECtert cells were incubated with increasing concentrations of heat shock protein inducers GGA (**A**) and HA15 (**B**), or chemical chaperones TUDCA (**C**) and sodium phenyl butyrate (**D**). The incubation was performed in serum-free medium for 72 h, followed by counting of Höchst-stained nuclei of attached cells. Dashed lines show levels from control (vehicle-treated) cells. * *p* ≤ 0.05, ** *p* ≤ 0.01, and *** *p* ≤ 0.001.

Heat shock proteins are universal guardians of proteostasis protecting cells from different types of cellular stress. In the mRNA expression experiments, we found that OxPAPC induced multiple HSPs. The most reproducible was induction by OxPAPC of HSPs belonging to the Hsp70 (HSPA) and Hsp40 (DNAJ) families (Table 2). In order to test if HSPs may play a role in the protective action of OxPAPC on serum-deprived cells, we applied low-molecular-weight inducers of HSPs acting through HSF1-dependent (geranylgeranylacetone) [31,32] and HSF1-independent (HA15 [33]) mechanisms. Both compounds mimicked the protective action of OxPAPC on ECs (Figure 8A,B). Furthermore, we tested chemical chaperones TUDCA and phenyl butyrate, which have physicochemical properties preventing protein denaturation [34]. These functional analogues of HSPs mimicked the protective action of OxPAPC on endothelial cells (Figure 8C,D). Thus, the maintenance of proteostasis by induction of HSPs is likely to explain, at least partially, the protection induced by OxPAPC. 

**Figure 9:**
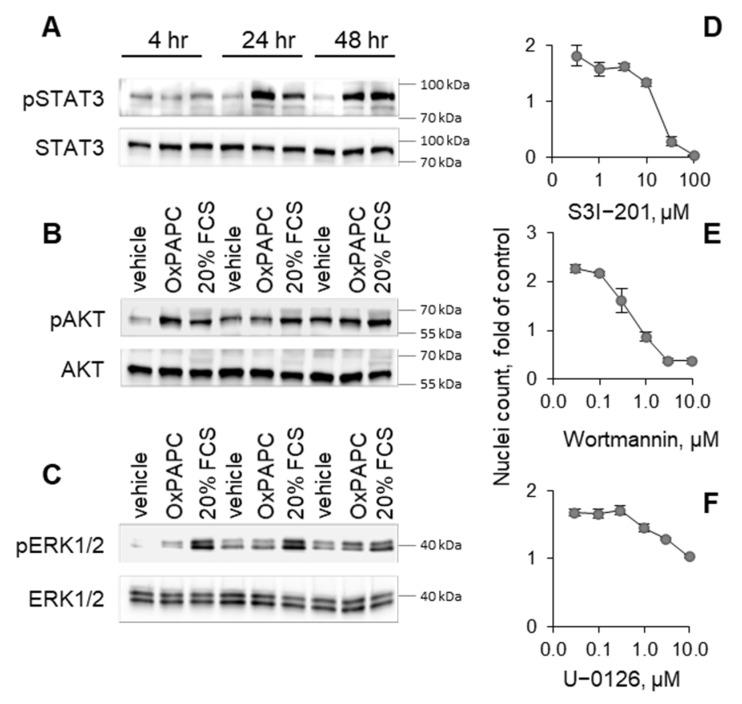
**OxPAPC induces cell stress tolerance via activation of RISK and SAFE pathways.** (**A**–**C**) HUVECtert cells were incubated with or without OxPAPC (20 µM) in serum-free medium for 48 h. Treatment with 20% serum was used as a control. Cells were harvested, proteins separated by SDS-PAGE gel, transferred to a nitrocellulose membrane and proteins were detected using specific antibodies. (**D**–**F**) Protective effect of OxPAPC (20 µM) against serum deprivation in HUVECtert cells was reversed by inhibitors of STAT3 (S3I−201), PI-3K/AKT (wortmannin), or MEK/ERK (U−0126F) as determined by Höchst-stained nuclei counting assay.

**Figure 10:**
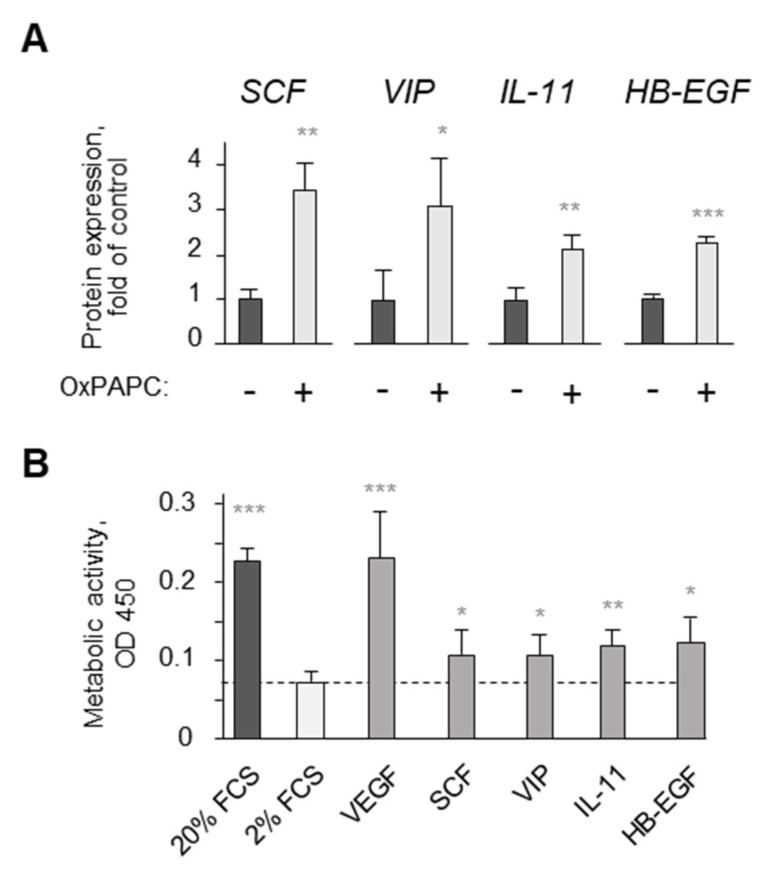
**OxPAPC induces expression of protective protein mediators in endothelial cells.** (**A**) HUVECs were incubated in medium 199 containing 2% serum and OxPAPC for 24 h, followed by ELISA for stem-cell factor (SCF), vasoactive intestinal peptide (VIP), interleukin-11 (IL-11), and heparin-binding EGF-like growth factor (HB-EGF). (**B**) Recombinant human proteins HB-EGF, vascular endothelial growth factor (VEGF), SCF, VIP or IL-11 were added to primary HUVEC in M199/2% serum. After 24 h, metabolic activity of remained cells was measured by XTT assay. Dashed line corresponds to control (vehicle-treated) samples. * *p* ≤ 0.05, ** *p* ≤ 0.01, and *** *p* ≤ 0.001.

Growth factors prevent cell death by activating protective pathways SAFE and RISK [35]. We found that OxPAPC stimulated key components of these survival pathways such as STAT3, AKT and ERK1/2 (Figure 9A–C). The treatment of cells with inhibitors of SAFE and RISK pathways reversed the protective action of OxPAPC (Figure 9D–F). The data allowed hypothesizing that OxPAPC stimulated the production of growth factors that protected cells through an autocrine mechanism. We studied this mechanism in HCAECs. Microarray analysis and RT-PCR have shown that OxPLs upregulated, in HCAECs, mRNAs encoding for growth factors (VEGF, heparin-binding epidermal growth factor, and stem cell factor), as well as protein mediators demonstrating cell-protective effects (interleukin 11 and vasoactive intestinal peptide) [36,37]. We found that these mediators were upregulated at the protein level by OxPLs and protected cells from serum deprivation (Figure 10A,B and [30,38]). Thus, the secretion of prosurvival growth factors and mediators likely represents an additional protective mechanism of OxPLs. 

### 3.3. Structure–Activity Relationship Analysis

#### 3.3.1. Oxidation of Fatty Acid Residues Is Crucial for Protective Activity

**Figure 11:**
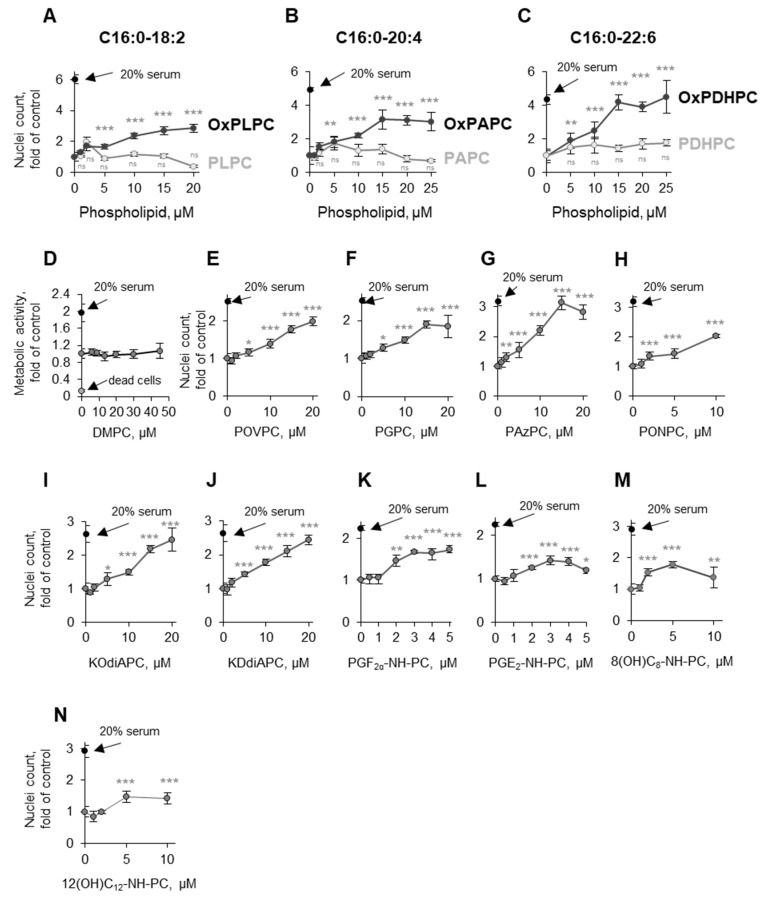
**Non-enzymatically oxidized PUFA-PLs or individual molecular species of OxPCs induce endothelial protection.** HUVECtert cells were incubated with increasing concentrations of OxPLPC or PLPC (**A**), OxPAPC or PAPC (**B**), OxPDHPC or PDHPC (**C**), as well as with non-oxidizable DMPC (**D**), or synthetic molecular species POVPC (**E**), PGPC (**F**), PAzPC (**G**), PONPC (**H**), KOdiAPC (**I**), KDdiAPC (**J**), or synthetic analogs PGF_2__α_-NH-PC (**K**), PGE_2_-NH-PC (**L**), 8(OH)C_8_-NH-PC (**M**), or 12(OH)C_12_-NH-PC (**N**) in medium 199 with 0% serum for 48 h, followed by quantification of metabolic activity (XTT assay) or counting Hoechst-labeled nuclei of attached cells. * *p* ≤ 0.05, ** *p* ≤ 0.01, and *** *p* ≤ 0.001.

In order to identify the structural characteristics of OxPLs that are important for their protective activity, we first compared the effects of *sn*-1-palmitoyl-phosphatidylcholines having different PUFAs at the *sn*-2 position, namely linoleic (18:2), arachidonic (20:4), and docosahexaenoic (22:6). It was found that all three phospholipids were protective, but only upon oxidation (Figure 11A–C). Because nonesterified oxidized arachidonic acid (OxAA) was not active (Figure 6F), one may conclude that a combination of a phospholipid scaffold with an oxidized fatty acid residue is an important determinant of protective activity.

We further tested several individual synthetic molecular species of OxPCs containing (i) oxidatively fragmented *sn*-2 residues of different length and (ii) different combinations of oxygen-containing groups. All of them, but not nonoxidizable saturated DMPC (Figure 11D), demonstrated protective activity comparable to that of OxPAPC (Figure 11E–J). In addition, we tested three phospholipase A_1_-/A_2_-resistant synthetic alkyl-amide OxPCs containing longer prostane cycle-containing or linear oxidized *sn*-2 residues [17]. Alkyl-amide OxPCs also protected cells; the effect was observed at the same or lower concentrations as compared to di-acyl-OxPLs, thus suggesting that OxPLs act as whole molecules and do not require cleavage to unmask their protective activity (Figure 11K–N).

**Figure 12:**
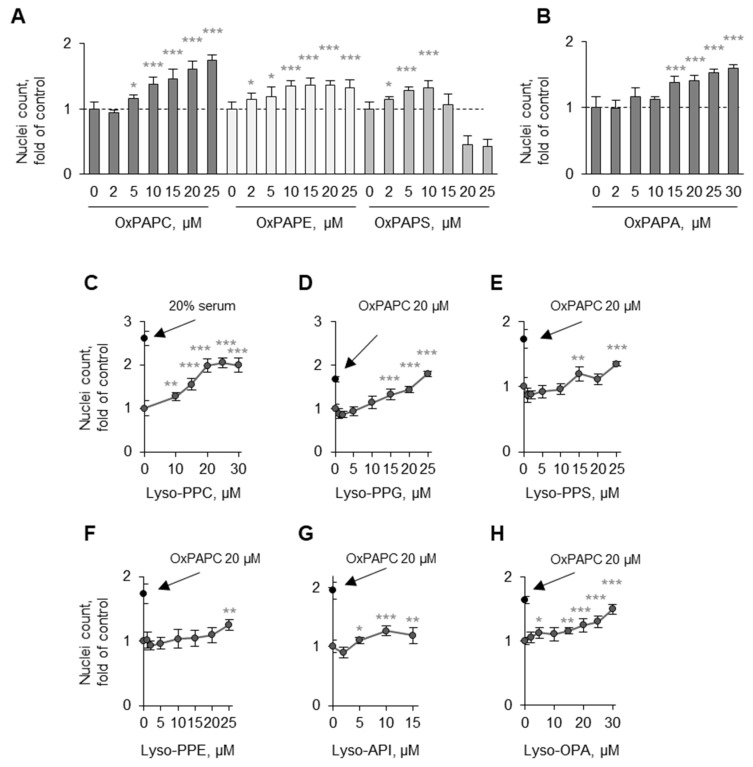
**Different classes of OxPLs and lysoPLs protect endothelial cells from serum deprivation.** HUVECtert cells were incubated with increasing concentrations of different classes of OxPLs (**A**,**B**) or lyso-PLs (**C**–**H**) in serum-free medium for 48 h, followed by quantification of survived attached cells by counting of Höchst-labeled nuclei. Dashed lines correspond to control (vehicle-treated) samples. * *p* ≤ 0.05, ** *p* ≤ 0.01, and *** *p* ≤ 0.001.

We further tested the role of a polar head group in the activity. The three most abundant classes of PLs, phosphatidylcholine, phosphatidylethanolamine, phosphatidylserine, as well as phosphatidic acid upon oxidation, demonstrated comparable activities, suggesting that the *sn*-3 group is not critical for the protective activity of OxPLs (Figure 12A,B).

#### 3.3.2. Lyso-PLs Are Protective

In contrast to normal (nonoxidized) PLs, OxPLs are rapidly degraded by abundantly expressed phospholipases A_2_, including PAF-acetylhydrolase, to lyso-PLs [39]. Lyso-PC is also generated from OxPCs nonenzymatically [40]. In human circulation, lyso-PLs are significantly more abundant than OxPLs and their total concentration is >100 µM [41]. We tested the effects of lyso-PLs on the survival of serum-deprived ECs. Lyso-PC, as well as other classes of lyso-PLs, demonstrated protective effects on ECs that were comparable with the action of OxPAPC (Figure 12C–H). Lysophosphatidic acid, which has no alcohol head group, was also protective. Taken together, the data of Figure 11A–C,E–N) and 12 suggest that protective activity is a characteristic of PL derivatives having either oxidized or missing *sn*-2 residue, while the groups at the *sn*-1 and *sn*-3 positions have minimal influence.

### 3.4. Detergent Properties as a Potential Prerequisite for the Protective Activity of OxPLs and Lyso-PLs

#### Upon Oxidation, PLs Acquire Detergent-Like Properties

**Figure 13:**
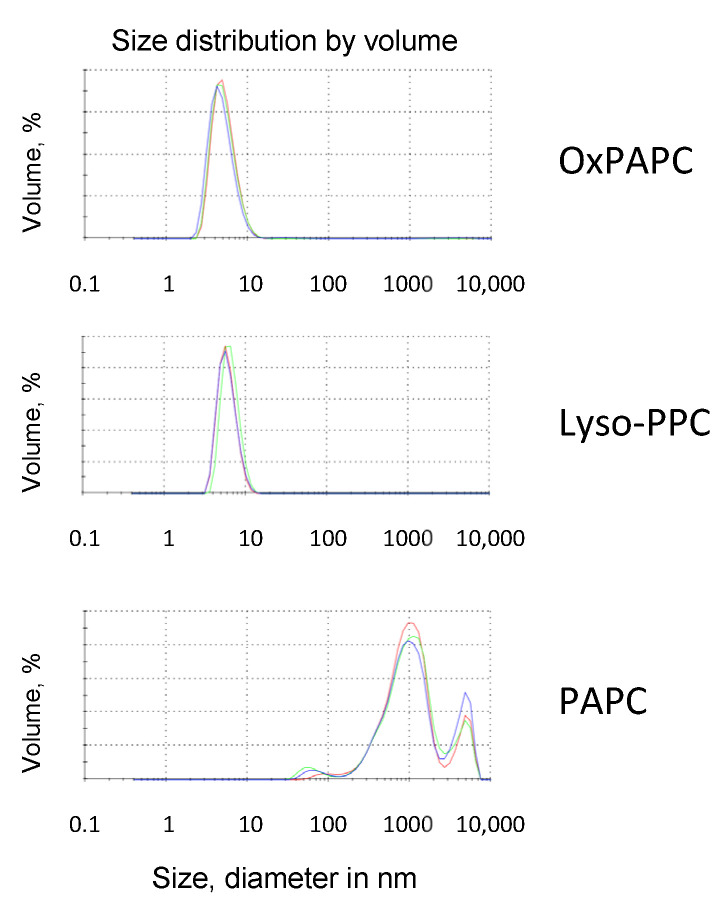
Particle size (volume-based size distribution) of OxPAPC, LysoPC and PAPC as measured by dynamic light scattering. Each measurement, including up to 18 sub-runs, was performed in triplicates using a Zetasizer Nano ZS instrument.

The analysis of different classes and molecular species of OxPLs described above suggests that, on the one hand, certain structural prerequisites for the protective activity of OxPLs do exist, namely an oxidized or missing *sn*-2 residue. On the other hand, the results clearly show that the structure–activity relationship is relatively promiscuous because a variety of classes and molecular species of OxPLs and lyso-PLs demonstrated comparable protective activity. A plausible explanation reconciliating these seemingly controversial findings is that upon the oxidation or formation of lyso-forms, PLs change their physicochemical properties and form supramolecular aggregates that are different from liposomes that are typical for nonoxidized PLs. This possibility is supported by the analysis of particles generated by OxPLs and their nonoxidized precursors under conditions that were used in our cell experiments. Using light-scattering analysis, we found that nonoxidized PAPC formed large aggregates that were 100 to >1000 nm in diameter (Figure 13). In contrast, OxPAPC and lyso-PC formed much smaller complexes, probably micelles, that were <10 nm in diameter (Figure 13). The data allow hypothesizing that the removal or shortening of the carbohydrate chain, or addition of polar oxygen-containing groups to the PUFA residues, imparts on PL molecules detergent-like properties. 

**Figure 14:**
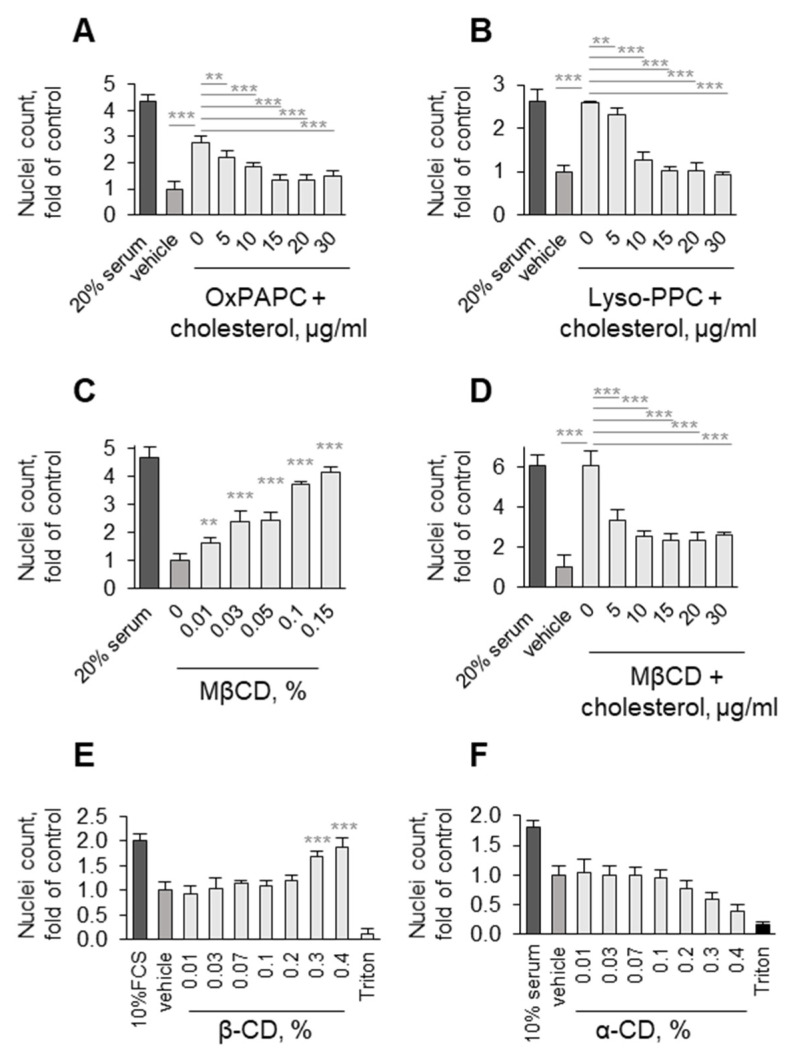
**Plasma membrane cholesterol seems to play a pivotal role in ability of lipids to protect cells from cell death**. (**A**,**B**) Increasing concentrations of cholesterol reverse protective action of OxPAPC (25 µM) and lysoPC (20 µM) on HUVECtert. (**C**) Cholesterol-binding methyl-β-cyclodextrin mimics protective action of OxPAPC and lysoPC. (**D**) Protective action of methyl-β-cyclodextrin (0.15 %) is reversed by co-treatment with cholesterol. (**E**,**F**) Ability of cyclodextrins to protect cells correlates with their cholesterol-binding capacity. Note that only cholesterol-binding β-cyclodextrin, but not inactive α-cyclodextrin was able to protect cells. ** *p* ≤ 0.01, and *** *p* ≤ 0.001.

At concentrations that are not lethal to cells, detergent-like molecules can influence cellular metabolism via changing different membrane characteristics, e.g., membrane fluidity, permeability, or microdomain organization. Furthermore, detergents can extract membrane proteins or lipids [42]. In support of the latter mechanism, OxPAPC and its components have been shown to extract cholesterol and change the microdomain organization of cell membranes [43,44]. We found that the addition of cholesterol reversed the protective effects of OxPAPC and lyso-PC (Figure 14A,B). Furthermore, β-cyclodextrin and methyl-β-cyclodextrin, which are well known to extract cholesterol from membranes [45], also protected cells (Figure 14C,E). In contrast, α-cyclodextrin, which consists of the same monomers as β-cyclodextrin but does not bind cholesterol due to the smaller size of the polymer cyclodextrin ring [45], had no protective activity (Figure 14F). Together with the data showing that, similarly to OxPAPC, also lyso-PC is capable of both extracting membrane cholesterol [46] and protecting ECs (Figure 12), our data allow hypothesizing that cholesterol extraction from cell membranes triggers events leading to cell protection, including the induction of intracellular and secreted prosurvival proteins described above.

## 4. Discussion

The major finding of this work is that OxPLs, which, similarly to other peroxidation products, are generally regarded as proinflammatory and toxic lipids, at low concentrations act on ECs protectively and mitigate biological and drug-induced stress.

The second important finding is an unexpected structure–activity relationship of the protective effect. Paradoxically, the effect of OxPLs on ECs appears to be a consequence of structural rearrangements in PL molecules that are usually regarded as being deleterious for biological membranes. Peroxidation or cleavage of an esterified PUFA producing hydrophilic or missing (lyso-PL) fatty acid residue led to the enhancement of micelle-forming properties as compared to nonoxidized PLs. This change in physicochemical parameters is usually regarded as a synonym of membrane toxicity. Counterintuitively, our experiments showed that the “detergent-like” properties of OxPLs did not impair their protective action, and probably were a prerequisite for this beneficial effect.

Detergents can influence different properties of biological membranes including the content or intramembrane distribution of cholesterol. Our results suggest that the protective action of low concentrations of Ox- and lyso-PLs may result from their known property to induce changes in membrane cholesterol [44,46]. This view is supported by the reversal of protective effects of OxPAPC and lyso-PC by cholesterol cotreatment. In addition, we described the protective action of cholesterol-binding cyclodextrins that are structurally very different from phospholipids. The action of methyl-β-cyclodextrin was also inhibited by cholesterol, thus further pointing to the redistribution or extraction of membrane cholesterol as a likely mechanism initiating the protective effects of OxPLs and lyso-PLs. A large body of experimental evidence, showing that the extraction of cholesterol stimulates the ligand-independent activation of “lipid raft”-associated growth factor receptors [47], points to this mechanism as a likely trigger of the protective effects of OxPLs, which will be analyzed in follow-up studies.

We identified several signaling pathways that are likely to work additively or synergistically in mediating the protective effect in endothelial cells. Our data point to the parallel upregulation of heat shock proteins, activation of the prosurvival SAFE and RISK pathways, as well as secretion of growth factors and protective peptide mediators, as likely mechanisms of protective action.

An important finding of the work is that the protection of cells is independent of electrophilic properties of OxPLs. Thus, the protective effect seems to be independent of classically activated electrophilic stress response, which is broadly recognized for its antioxidant and cell-protective action [48]. 

The potential biological importance of our findings is emphasized by the fact that ECs represent the major noncirculating cell type directly and permanently contacting oxidized and lyso-PLs in circulation and atherosclerotic lesions. Total concentrations of OxPLs locally in atheroma can achieve micromolar range [14], which is comparable with concentrations inducing protective effects in cultured ECs in our experiments. Moreover, circulating total concentrations of lyso-PLs in humans are >100 µM [49]. Thus, both oxidized and lyso-PLs are present in vivo at concentrations sufficient to induce protective biological reactions in endothelial cells. 

Recent scanning electron microscopy data show that atherosclerotic plaques demonstrate morphological signs of endothelial regeneration [50]. It is tempting to speculate that OxPLs, that are abundant in plaques [14,51], may represent one of the factors modulating the dynamic equilibrium between the degeneration and regeneration processes. In addition to better understanding the biology of a healthy and atherosclerotic endothelium, analysis of the protective effects of OxPLs is of potential practical relevance. Endothelial toxicity is an important adverse effect of (i) cytostatics, (ii) other classes of low-molecular-weight drugs, (iii) biologics, as well as (iv) radiation therapy [52,53,54]. Thus, a better understanding of humoral mechanisms regulating the live/death balance of endothelial cells potentially can provide new hints for the protection of the endothelium against iatrogenic damage.

Our findings of the protective action of low concentrations of OxPLs further characterize these lipids as an illustration of the Paracelsus’ maxim “only the dose makes the poison”. We have shown previously that OxPLs induced anti-inflammatory effects at low concentrations but, in the same cell type, became proinflammatory at high concentrations [14]. In a similar way, a biphasic biological effect has been described for OxPLs containing esterified cyclopentenone prostaglandin [7]. In this study, we observed a biphasic action of OxPLs in respect to toxicity in ECs. This effect is in good agreement with the previously described hormetic action of OxLDL and lipid peroxidation products (reviewed in [55,56]). Further analysis of the mechanisms underlying the protective hormetic action of OxPLs will add to a better understanding of how circulating phospholipids regulate endothelial health. 

## 5. Conclusions

In summary, our experimental results suggest that OxPLs trigger in endothelial cells a sequence of events resulting in increased cellular-stress tolerance. Oxidation or cleavage of an esterified PUFA changes the physicochemical properties of phospholipids, making them capable of modulating membrane cholesterol content or distribution. Membrane cholesterol redistribution is followed by the induction of downstream protective mechanisms including chaperones and extracellular-protein mediators. Further analysis of the mechanisms and physiological relevance of the protective effects of OxPLs on the endothelium is in process.

## 6. Patents

The results of this study are partially included into patent WO2019025324 (A1).

## Figures and Tables

**Table 1:** Primary antibodies (all from Cell Signaling) that were used for Western blotting.

Antibody	Dilution	Protein Size, kDa	Host
α-actin	1:3000	42	mouse
α-AKT	1:2000	60	mouse
α-phospho-AKT	1:1000	60	rabbit
α-ERK1/2	1:1000	42	rabbit
α-phospho-ERK1/2	1:2000	42	rabbit
α-STAT3	1:1000	80	mouse
α-phospho- STAT3	1:2000	90	rabbit
α-HO-1	1:100	33	mouse

## Data Availability

Data is contained within the article.

## Abbreviations

8(OH)C_8_-NH-PC	1-*O*-hexadecyl-2-deoxy-2-(8-hydroxy-octanoyl)amino-*sn*-glycero-3-phosphocholine
12(OH)C_12_-NH-PC	1-*O*-hexadecyl-2-deoxy-2-(12-hydroxy-dodecanoyl)amino-sn-glycero-3-phosphocholine
8-iso-PGA_2_	8-iso-prostaglandin A_2_
α-CD	α-cyclodextrin
ara-C	cytosine β-D-arabinofuranoside hydrochloride, arabinoside-C
BCH	2-aminobicyclo[2.2.1]heptane-2-carboxylic acid, 2-amino-2-norbornanecarboxylic acid
BHA	butylated hydroxyanisole
tBuOOH	*tert*-butyl hydroperoxide
β-CD	β-cyclodextrin
CM	conditioned medium
CYC	cytochrome c
DMPC	1,2-dimyristoyl-*sn*-glycero-3-phosphocholine
EC	endothelial cell
FBS	fetal bovine serum
FCS	fetal calf serum
GGA	geranylgeranylacetone
HBMVEC	human bovine microvascular endothelial cell
HB-EGF	heparin-binding EGF-like growth factor
HCAEC	human coronary artery endothelial cell
HLMVEC	human lung microvascular endothelial cell
HMGB1	high-mobility group B1 protein
HPAEC	human pulmonary artery endothelial cell
HSF1	heat-shock factor 1
HSP	heat-shock protein
HUVEC	human umbilical vein endothelial cell
IL-11	interleukin-11
KDdiAPC	1-palmitoyl-2-(4-keto-dodec-3-ene-dioyl)-*sn*-glycero-3-phosphocholine
KOdiAPC	1-palmitoyl-2-(5-keto-6-octene-dioyl)-*sn*-glycero-3-phosphocholine
LDH	lactate dehydrogenase
LDHA	lactate dehydrogenase A chain
LDHB	lactate dehydrogenase B chain
Lyso-PPC	1-palmitoyl-2-hydroxy-*sn*-glycero-3-phosphocholine
Lyso-PPE	1-palmitoyl-2-hydroxy-*sn*-glycero-3-phosphoethanolamine
Lyso-PPG	1-palmitoyl-2-hydroxy-*sn*-glycero-3-phosphoglycerol
Lyso-OPA	1-oleoyl-2-hydroxy-*sn*-glycero-3-phosphate
Lyso-PPI	1-palmitoyl-2-hydroxy-*sn*-glycero-3-phosphoinositol
Lyso-PPS	1-palmitoyl-2-hydroxy-*sn*-glycero-3-phosphoserine
MβCD	methyl-β-cyclodextrin
NAC	N-acetyl cysteine
OxAA	oxidized arachidonic acid
OxPAPA	oxidized 1-palmitoyl-2-arachidonoyl-*sn*-glycero-3-phosphatidic acid
OxPAPC	oxidized 1-palmitoyl-2-arachidonoyl-*sn*-glycero-3-phosphocholine
OxPAPE	oxidized 1-palmitoyl-2-arachidonoyl-*sn*-glycero-3-phosphoethanolamine
OxPAPG	oxidized 1-palmitoyl-2-arachidonoyl-*sn*-glycero-3-phosphoglycerol
OxPAPS	oxidized 1-palmitoyl-2-arachidonoyl-*sn*-glycero-3-phosphoserine
OxPDHPC	oxidized 1-palmitoyl-2-docosahexaenoyl-*sn*-glycero-3-phosphocholine
OxPLPC	oxidized 1-palmitoyl-2-linoleoyl-*sn*-glycero-3-phosphocholine
OxPL	oxidized phospholipid
PAF	platelet-activating factor
PAzPC	1-palmitoyl-2-azelaoyl-*sn*-glycero-3-phosphocholine
PAPA	1-palmitoyl-2-arachidonoyl-*sn*-glycero-3-phosphatidic acid
PAPC	1-palmitoyl-2-arachidonoyl-*sn*-glycero-3-phosphocholine
PAPE	1-palmitoyl-2-arachidonoyl-*sn*-glycero-3-phosphoethanolamine
PAPG	1-palmitoyl-2-arachidonoyl-*sn*-glycero-3-phosphoglycerol
PAPS	1-palmitoyl-2-arachidonoyl-*sn*-glycero-3-phosphoserine
PGA_2_	prostaglandin A_2_
PGE_2_-NH-PC	1-*O*-hexadecyl-2-deoxy-2-(prostaglandin E_2_)amino-*sn*-glycero-3-phosphocholine
PGF_2α_-NH-PC	1-*O*-hexadecyl-2-deoxy-2-(prostaglandin F_2α_)amino-*sn*-glycero-3-phosphocholine
PGK1	phosphoglycerate kinase 1
PGPC	1-palmitoyl-2-glutaroyl-*sn*-glycero-3-phosphocholine
PONPC	1-palmitoyl-2-(9’-oxononanoyl)-*sn*-glycero-3-phosphocholine
POVPC	1-palmitoyl-2-(5’-oxovaleroyl)-*sn*-glycero-3-phosphocholine
PUFA	polyunsaturated fatty acid
RIPA	radioimmunoprecipitation assay
SCF	stem-cell factor
TER	transendothelial electrical resistance
TUDCA	tauroursodeoxycholic acid
VEGF	vascular endothelial growth factor
VIP	vasoactive intestinal peptide
